# A benchmark dataset of electrical signals from a permanent magnet synchronous generator for condition monitoring

**DOI:** 10.1016/j.dib.2025.112040

**Published:** 2025-09-09

**Authors:** Rafael Noboro Tominaga, Santiago Silveira Barbosa, Luan Andrade Sousa, Angelo dos Santos Lunardi, Rodolfo Varraschim Rocha, Sérgio Luciano Ávila, Bruno Souza Carmo, Renato Machado Monaro, Maurício Barbosa de Camargo Salles

**Affiliations:** a University of São Paulo (USP), São Paulo, Brazil; b Federal University of Mato Grosso (UFMT), Cuiabá, Brazil; c Federal Institute of Santa Catarina (IFSC), Florianópolis, Brazil

**Keywords:** Power generator, Electrical currents, Signature analysis, Behavior identification, Fault detection

## Abstract

The proper monitoring of sensitive components in rotating electrical machines plays a critical role in preventing internal faults that may lead to irreversible damage and unplanned shutdowns. Offshore wind power generation is increasingly adopting permanent magnet synchronous generators (PMSGs) because of their high efficiency and low maintenance requirements. However, internal short-circuit faults remain a challenge and require effective fault detection strategies. Inter-turn and inter-winding faults, in particular, may not cause immediate damage but can evolve over time, leading to severe equipment failures. These failures may require generator shutdowns, resulting in significant financial and operational losses. This dataset provides high-resolution electrical measurements from a PMSG under healthy and faulty conditions, supporting the development and validation of related diagnostic and control strategies.

We collected data from a laboratory test bench that allows controlled insertion of internal faults, such as short-circuits between turns and windings. The generator, connected to the grid via a power converter, was monitored using an Imperix B-Box RCP system loaded with control algorithms developed in Simulink. Signals were sampled at 20 kHz and recorded through the Imperix Cockpit, with each test lasting three seconds and capturing pre-fault, fault, and post-fault conditions. This structure enables users to study transient responses, steady-state behavior with faults, and system recovery.

The dataset comprises 225 .mat files covering 24 fault cases and one healthy case, each tested under three torque levels and three rotational speeds. The selected operating conditions reflect typical points of a 15-megawatt offshore wind turbine. In addition to the raw data, the dataset includes a Python interface to facilitate visualization. The dataset can support diverse applications, such as validating analytical models of PMSGs, benchmarking fault detection algorithms, and generating synthetic data for further testing. It may also serve as a practical tool in electrical engineering education, especially in courses focused on wind energy systems and fault analysis.

Specifications Table

**Table:** 

Subject	Energy Engineering and Power Technology; Electrical and Electronic Engineering; Machine Design; Data Engineering; Signal Processing.
Specific subject area	Analysis of the electrical current signatures of a permanent magnet synchronous generator
Type of data	Table, Figure Raw data, not analysed, not filtered, and not processed. Processed data can be obtained by direct request to the authors
Data collection	Electro-electronic industrial sensors installed at key positions collected the variables of interest. The section's experimental design, materials and methods show the instrumentation used. A data acquisition device manages the sensor signals and generates the dataset files. Two people conducted the tests together, even with a script automating test execution, and labeled the files according to the test conditions.
Data source location	Escola Politécnica, University of São Paulo, São Paulo, Brazil
Data accessibility	Repository name: PMSG-3phase-Dataset Data identification number: 10.5281/zenodo.15741561 Direct URL to data: https://github.com/InnovaPower/MitDev-Eletrica Instructions for accessing these data: documentation in the same GitHub.
Related research article	None.

## 1 Value of the Data

• Analytical models of variable-speed synchronous generators under healthy and faulty conditions can be validated using the laboratory data available here.
• This dataset serves as a benchmark for evaluating and comparing new data-driven algorithms, relying on public and reliable data sources.
• Synthetic data can be generated to reproduce patterns and characteristics of this dataset. The idea is to use the dataset to validate generative models, which can then be adapted to extrapolate data to other operational conditions and nominal power levels not originally covered.
• This dataset helps mitigate overfitting in machine learning algorithms designed to detect internal faults by augmenting the training data.
• The dataset can be used in engineering education to illustrate the effects of internal failures in generators, as well as in training machine learning algorithms and validating mathematical models and simulations.

## 2 Background

Wind energy has emerged as one of the most prominent renewable sources in the global transition toward cleaner and more sustainable power generation [1]. This study focuses on the PMSG because of its widespread use in modern power systems, where ensuring reliability and operational safety has become a critical concern. Since different machine topologies exhibit unique behaviors when subjected to the same type of fault, analyzing the PMSG provides a meaningful starting point for understanding short-circuit dynamics in electrical machines. By detecting failures early, effective condition monitoring prevents costly damage and downtime. In this context, signal processing techniques, such as filtering methods, combined with artificial intelligence (AI) approaches, have shown promising results in identifying abnormal behavior in wind turbine components [2]. However, performing these techniques depends on the availability of reliable and representative datasets for training and validation. Short-circuit faults, such as inter-turn and inter-winding faults, are challenging to detect, as they often present subtle electrical signatures in the early stages, despite their potential to evolve into more serious conditions if left unaddressed. To tackle this issue, the present work proposes a dedicated dataset designed to support the development and evaluation of intelligent monitoring systems for fault detection in wind turbine generators.

## 3 Data Description

The study of inter-turn and inter-winding short-circuits in a permanent magnet synchronous generator (PMSG) prompted the tests. Each recording has a time window of three seconds.

In the fault tests, the first second captures the machine operating under steady-state conditions, without any disturbances. This initial period is crucial to establish a baseline of normal machine behavior. By confirming the machine’s healthy state before fault insertion, it becomes possible to attribute subsequent deviations in the recorded measurements to the fault itself, thereby ensuring the credibility of the dataset.

**Fig. 1:**
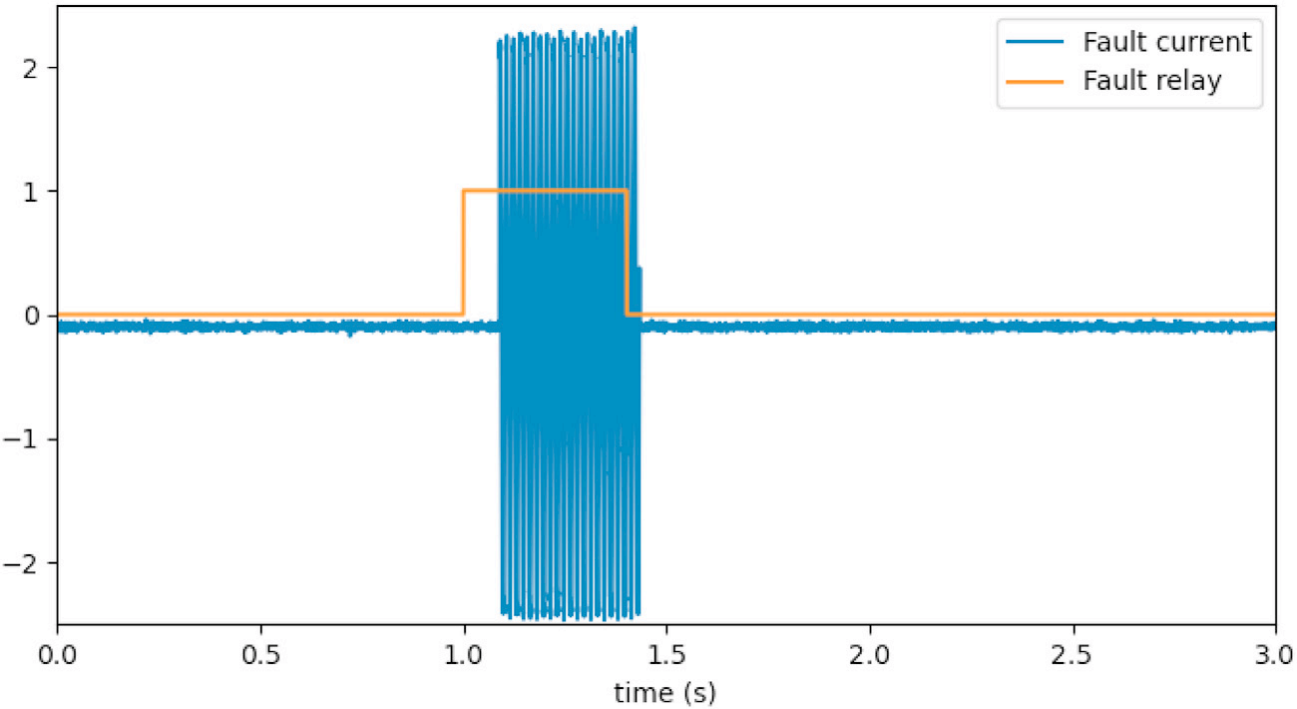
Fault current delay and stages overview.

The test system introduces the fault during the following 400 ms. Fig. 1 illustrates the brief delay caused by the relay’s response time required for the fault current to develop. The fault relay is a Boolean variable, and a sensor measures fault current in amperes. This delay appears when the time interval between the activation of the fault relay and the onset of oscillations in the fault current. This time window also allows the observation of both the fault insertion transient and the machine behavior in steady-state operation under fault conditions.

The rest of the window captures the fault removal and shows how the system returns to normal operation. Thus, the window encompasses four distinct stages: normal operation, fault insertion, faulted operation, and fault removal. This configuration supports various analyses, such as transient detection, faulted-state characterization, and pre- and post-fault assessment.

The dataset comprises 225 .mat extension files, generated from tests of 24 different short-circuit fault conditions, along with one healthy operating condition. Each short-circuit and healthy case resulted in nine operating scenarios, formed by combinations of three mechanical speeds and three torque levels. The dataset includes only inter-turn and inter-winding short-circuit faults. Other short-circuit types, such as inter-phase faults, were deliberately excluded because their high severity poses a greater risk of permanent damage to the machine. Industrial protection systems and diagnostic methods commonly studied in the literature can detect this high severity more easily, making their acquisition less relevant for research. Reproducing such faults would require a more robust and costly experimental setup, which discourages the construction of dedicated test benches because of the increased risk of permanent equipment damage.

**Fig. 2:**
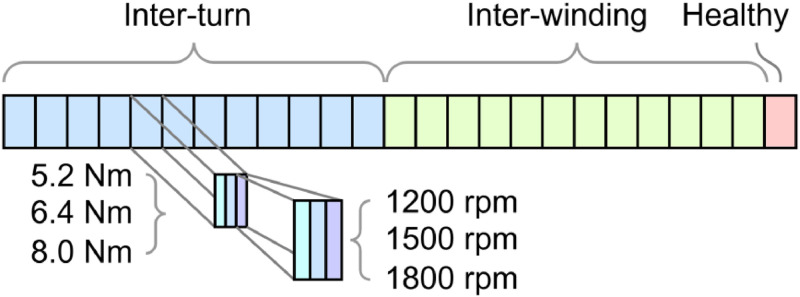
Fault current delay and stages overview.

**Fig. 3:**
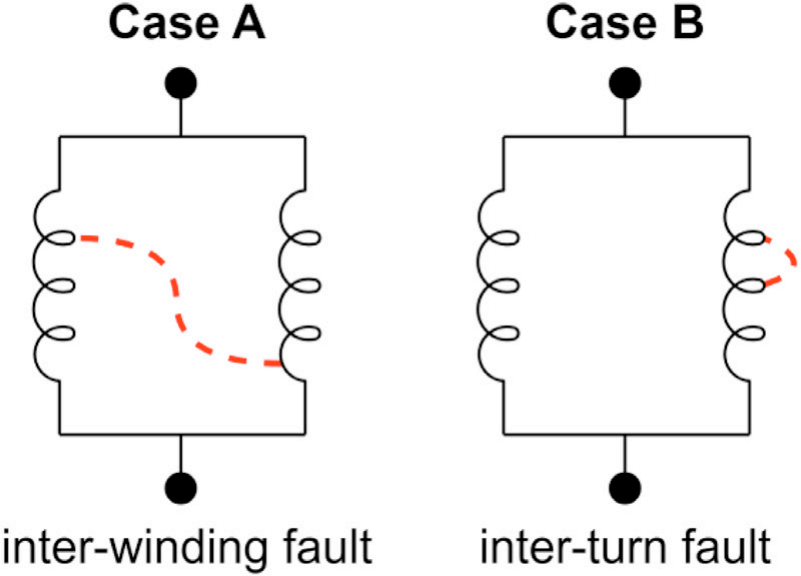
Fault cases considered for the PMSG.

Fig. 2 helps to better understand these files, where each of the 25 rectangles represents one of the 24 short-circuit fault conditions, along with the healthy case. Fig. 3 presents the faults considered and their corresponding connection, with the location of the applied short-circuit highlighted in red dashed lines.

Each file is approximately 5.2 MB in size, resulting in about 1.2 GB of data. The selection of the .mat format ensured efficient data compression, helping reduce storage requirements. In addition, although it is associated with proprietary software (MATLAB), this format is widely supported by free tools such as the SciPy [3] library in Python, which allows users to access the dataset without a MATLAB license.

Another advantage of using the .mat format is the structured organization it provides, which simplifies the data loading process. Unlike plain text formats such as .csv, users do not have to write custom parsing routines, making the dataset more immediately usable.

Besides the 225 .mat files, the dataset includes two supplementary files that are part of a lightweight graphical interface designed to allow quick previewing of the data, requiring no custom implementation. Although not designed for advanced data analysis, this interface provides a quick initial visualization of the signals.

The file dataset_preview.py, implemented in Python 3.12.7, contains the interface. To run it, the following modules must be installed: os, sys, NumPy (tested with 1.26.4), SciPy (1.13.1), Matplotlib (3.9.2), and PyQt5 (5.15.10). The file dxx_position.png is also required for the interface to function correctly, as it provides the layout reference for the machine’s winding configuration. This work provides further details on the interface in a later section.

### 3.1 Features description

Table 1 presents 32 variables, each detailed with its index, name, description, and measurement unit. There are no missing values.

**Table 1:** Description of the features.

Column	Feature	Description	Unit
1	t	Time elapsed after the start of the recording.	s
2	Ia	Current measured in phase A at the generator output terminals.	A
3	Ib	Current measured in phase B at the generator output terminals.	A
4	Ic	Current measured in phase C at the generator output terminals.	A
5	Id	Direct-axis current of the generator calculated according to current measurements.	A
6	Iq	Quadrature-axis current of the generator calculated according to current measurements.	A
7	Idref	Direct-axis current reference imposed.	A
8	Iqref	Quadrature-axis current reference imposed according to speed measurements.	A
9	Ifault	Fault current measured.	A
10	Va	Voltage in phase A measured at the point between the generator and the converter.	V
11	Vb	Voltage in phase B measured at the point between the generator and the converter.	V
12	Vc	Voltage in phase C measured at the point between the generator and the converter.	V
13	Va_ref	Reference voltage calculated in phase A for converter output.	V
14	Vb_ref	Reference voltage calculated in phase B for converter output.	V
15	Vc_ref	Reference voltage calculated in phase C for converter output.	V
16	Vd	Direct-axis voltage of the generator calculated according to voltage measurements.	V
17	Vq	Quadrature-axis voltage of the generator calculated according to voltage measurements.	V
18	Vd_conv	Direct-axis voltage calculated for converter output.	V
19	Vq_conv	Quadrature-axis voltage calculated for converter output.	V
20	Vdc	Voltage on the DC-link of the converter.	V
21	Action_pi_d	Calculated PI controller action for direct-axis.	V
22	Action_pi_q	Calculated PI controller action for quadrature-axis.	V
23	Torq_ele	Calculated Electric torque.	Nm
24	Torq_mec	Measured Mechanical torque.	Nm
25	Theta_est	Encoder position estimated with a Kalman filter [4].	rad
26	Theta_mea	Encoder position measured.	rad
27	Spd	Rotor speed measured.	rad/s
28	Fault_Relay	Fault relay command. A high level indicates that the test script has issued the command to close the contactor and start the short-circuit fault. This work provides further details about the test script in a later section.	-
29	Da	Phase A converter duty cycle.	-
30	Db	Phase B converter duty cycle.	-
31	Dc	Phase C converter duty cycle.	-
32	Package_loss	Package loss of CAN communication. This variable corresponds to a discrete integrator that increments continuously until successful communication occurs, after which it resets. Higher values indicate greater delays or interruptions in the CAN communication.	-

**Fig. 4:**
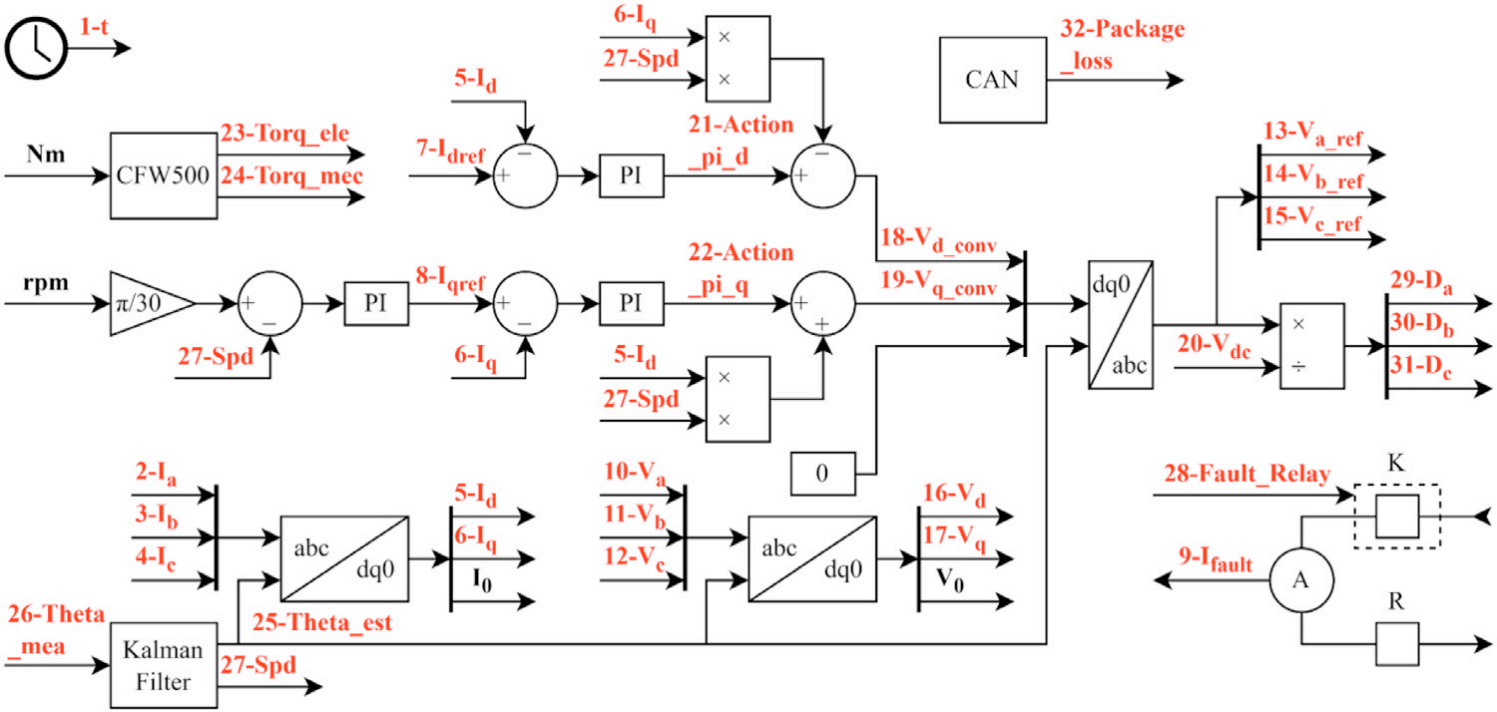
PMSG control and location of variables.

The variables are ordered according to their unit type to facilitate interpretation. The dataset includes both directly measured signals, acquired through physical sensors, and derived quantities, obtained through post-processing or estimation. All signals were sampled at a rate of 20,000 points per second, ensuring high temporal resolution for transient and steady-state analysis. Fig. 4 illustrates the location of each variable within the PMSG control diagram, offering a visual reference for their role and position in the system.

### 3.2 Explanation of filename composition

The dataset filenames encode all relevant test information, eliminating the need for additional documentation. Each filename combines prefixes and suffixes that specify the fault type, terminal location, resistance, speed, and torque. This naming convention simplifies data navigation and analysis by making test conditions immediately identifiable.

**Fig. 5:**
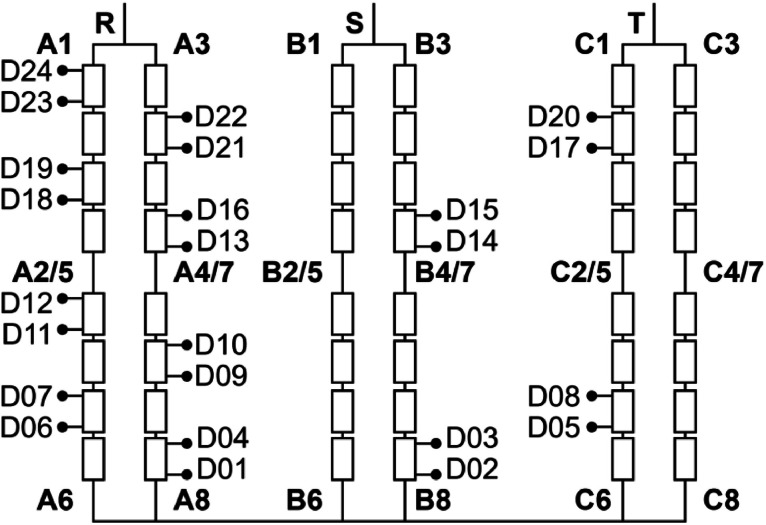
Explanation of the derivations of the windings.
• R026 denotes the fault resistance in tenths of ohms; accordingly, all tests used a resistance of 2.6 Ω.
• S1200 represents the rotational speed in revolutions per minute (rpm). The speed values adopted for testing were 1200, 1500, and 1800 rpm.
• T52 shows the mechanical torque in tenths of newton-meters. The torque values adopted for testing were 5.2, 6.4, and 8.0 Nm.

As an example, consider the file named FAULT_WINDINGS_D16_D18_R026_S1200_T52. In this case:

• FAULT indicates a fault; alternatively, the prefix HEALTHY is used for data corresponding to normal (non-faulty) operating conditions.
• When a fault is present, the following field indicates its type. This dataset includes two fault types: TURNS (a short-circuit between turns of the same winding) and WINDINGS (a short-circuit between different windings).
• D16_D18 refers to the fault insertion points, corresponding to specific terminals along the windings. Fig. 5 shows the physical location of all 24 terminals, labeled from D01 to D24.

All test combinations are unique, with no repetitions conducted throughout the experiments. The selected speed and torque values represent the typical operating points of a 15-megawatt offshore wind turbine [5]. This type of turbine typically operates between 5 and 7.55 rpm, where 7.55 rpm corresponds to the rated speed. Accordingly, the generator on the test bench was set to 1800 rpm, which matches its nominal speed. By proportional scaling, the equivalent minimum speed is approximately 1200 rpm. Torque values were defined analogously, resulting in operating points ranging from 5.2 to 8.0 Nm. For reference, the 15-megawatt offshore wind turbine has a nominal torque of 21 MNm.

There are three typical modes of wind turbine operation: at low wind speeds, torque control keeps the rotor turning at a minimum speed; at moderate wind speeds, torque control adjusts the rotor speed to maintain an optimal tip-speed ratio; and at high wind speeds, pitch control is used to keep the rotor speed at its rated value [5]. The present dataset focuses exclusively on the second mode. This operating region is particularly relevant because wind turbines typically spend most of their active time in this mode, and it is within this range that maximum power extraction is achieved.

### 3.3 Dataset graphical interface

**Fig. 6:**
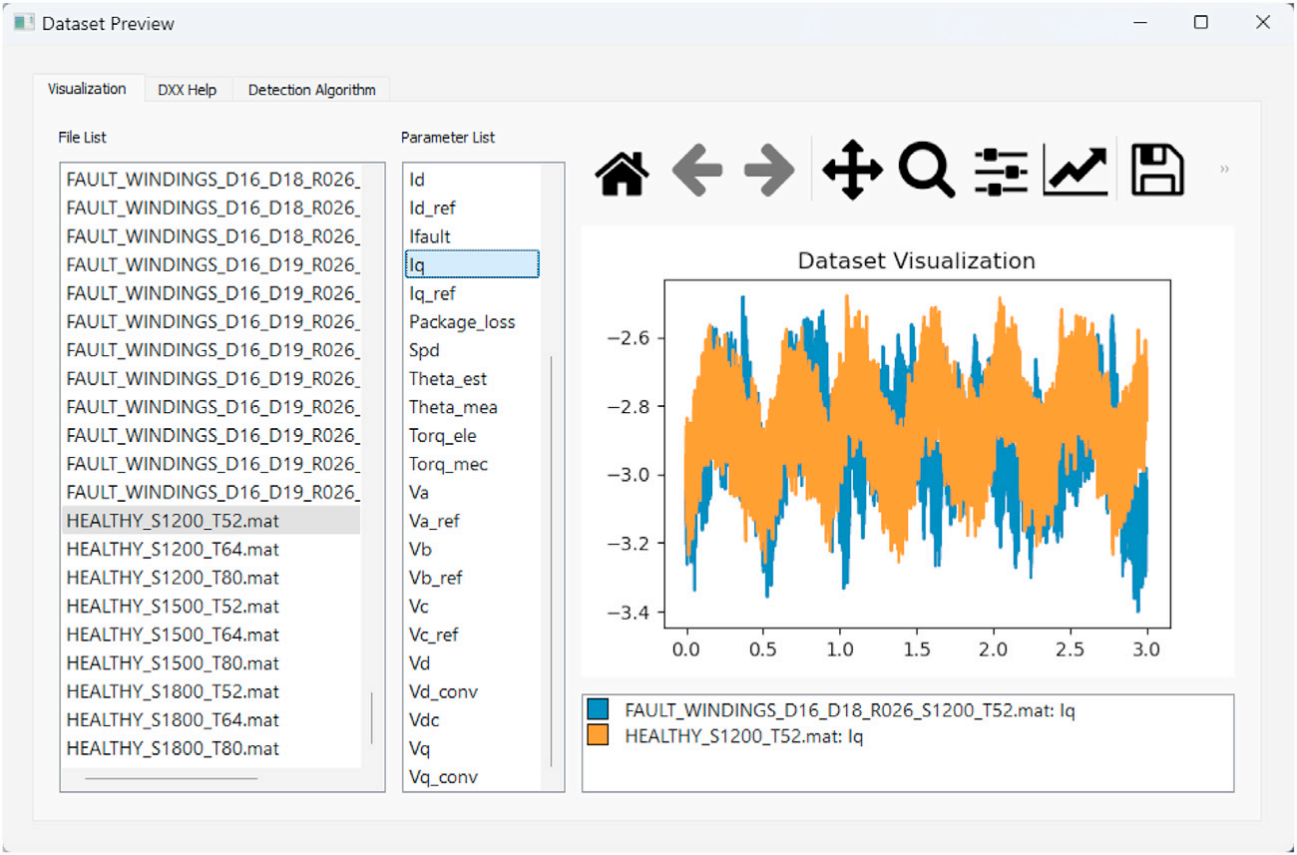
Visualization tab of dataset preview.

**Fig. 7:**
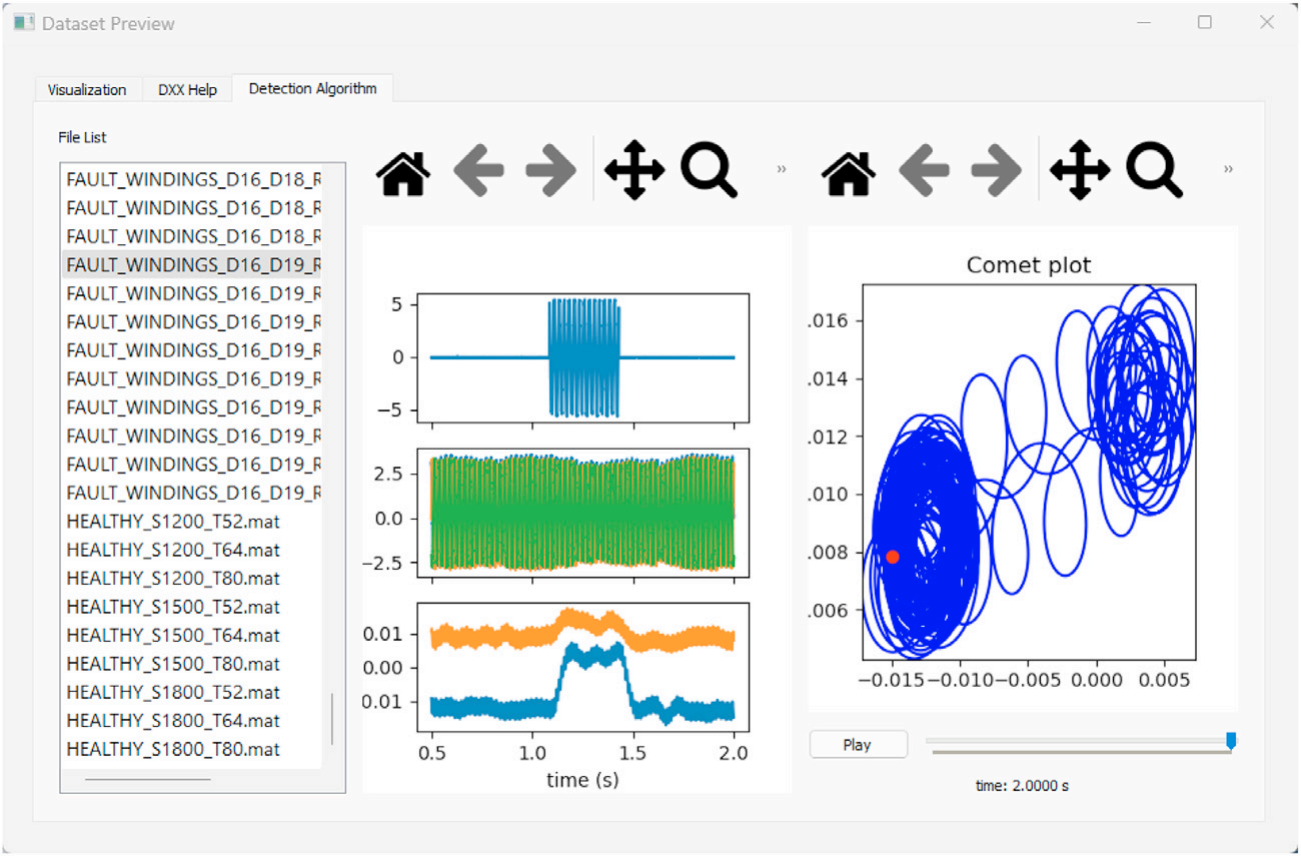
Detection algorithm tab of dataset preview.

Returning to the graphical interface introduced earlier, a more detailed description follows. After outlining the dataset structure and the test conditions, Fig. 6, Fig. 7 illustrate two of the three tabs available in the interface, each designed to assist users in exploring and interpreting the data more efficiently.

The first tab, shown in Fig. 6, contains three columns: the list of data files, the list of variables available in each file, and a plot area. This layout enables the user to select and visualize multiple variables from different files simultaneously, with no restriction on the number of curves plotted. The graph supports interactive features, including zooming and exporting the image. The graphical interface allows generating Fig. 1 using the export feature. All plotted data are raw signals, with no filtering or processing applied.

The second tab displays the PMSG winding layout diagram, previously presented as Fig. 5. This visual reference enables quick consultation of the positions of the derivation points along the windings, assisting the user in interpreting the naming conventions and test configurations.

The third tab, shown in Fig. 7, incorporates a fault detection algorithm developed by Rocha [6]. The algorithm applies a low-pass filter to the negative-sequence current obtained via the DQ0 transformation, generating an indicator that is sensitive to internal asymmetries caused by faults. Although this algorithm is not intended to support analytical conclusions within the scope of this work, it is included as a visual aid to provide the user with a preliminary notion of how faults affect the generator's behavior. In particular, the algorithm can detect medium- and high-severity short-circuits based on the displacement of the Comet plot during the time interval while the fault current is present. This simple approach not only supports visual interpretation but also opens the possibility for future studies aiming to enhance sensitivity and detect lower-severity faults.

This tab comprises three columns: the first for file selection; the second for plotting the fault current, the PMSG phase currents (A, B, and C), and the output of the detection algorithm; and the third for displaying a Comet plot that visualizes the evolution of the fault indicator throughout the signal. The interface also allows for stepwise playback of the signal progression, allowing users to observe the dynamic behavior of the indicator.

## 4 Experimental Design, Materials and Methods

After summarizing the dataset, the following section presents detailed information about the components used in the test bench assembled with the permanent magnet synchronous generator (PMSG), as well as the data acquisition process.

The PMSG converts mechanical energy into electrical energy through the interaction between a rotating magnetic field and stationary windings. Table 2 presents the main design specifications of the PMSG. The generator design features 24 winding derivations, each located at a specific relative distance from the neutral point to ease access to the coils. These relative distances, expressed as percentages, are listed in Table 3.

**Table 2:** PMSG specification.

Characteristic	Value
Manufacturer	Equacional [7]
Power	2.5 kVA
Armature voltage	230 V - three-phase line voltage
Armature current	6.3 A (YY)
Nominal speed	1800 rpm at 60 Hz
Thermal insulation class	F - 155 °C
Regime of work	S1 (constant load)
Number of poles	4

**Table 3:** Winding derivations characteristics description.

Terminal	Phase	Distance (%)
D01	A	0.460
D02	B	2.310
D03	B	10.000
D04	A	12.500
D05	C	12.900
D06	A	14.800
D07	A	22.200
D08	C	25.000
D09	A	29.600
D10	A	32.400
D11	A	42.200
D12	A	44.900
D13	A	50.000
D14	B	52.300
D15	B	59.700
D16	A	61.600
D17	C	62.500
D18	A	64.800
D19	A	72.200
D20	C	74.100
D21	A	78.600
D22	A	82.400
D23	A	92.100
D24	A	94.900

The experimental test bench, designed to capture data under both healthy and faulty conditions, has two generators: a squirrel cage induction generator and a PMSG, both driven by a WEG squirrel cage induction motor. The PMSG is among the most widely used generators in wind turbines for full-power conversion and variable-speed operation, because of several advantages such as high efficiency, high reliability [8], high power density, and low maintenance requirements when compared to induction generators [9]. Other generator types, such as fixed-speed and variable-speed synchronous generators, have also been previously tested under healthy and faulty conditions, with their corresponding datasets made publicly available [10].

The test bench's PMSG operates in a three-phase double-star (YY) configuration. However, other connection arrangements are also possible, such as symmetric six-phase (Y), asymmetric six-phase (Y), and symmetric twelve-phase (Y) configurations. The machine generates electricity through electromagnetic induction, using permanent magnets on the rotor to create a rotating magnetic field that induces AC voltages in the stator windings. It operates synchronously, meaning the rotor speed directly determines the output frequency [11].

**Fig. 8:**
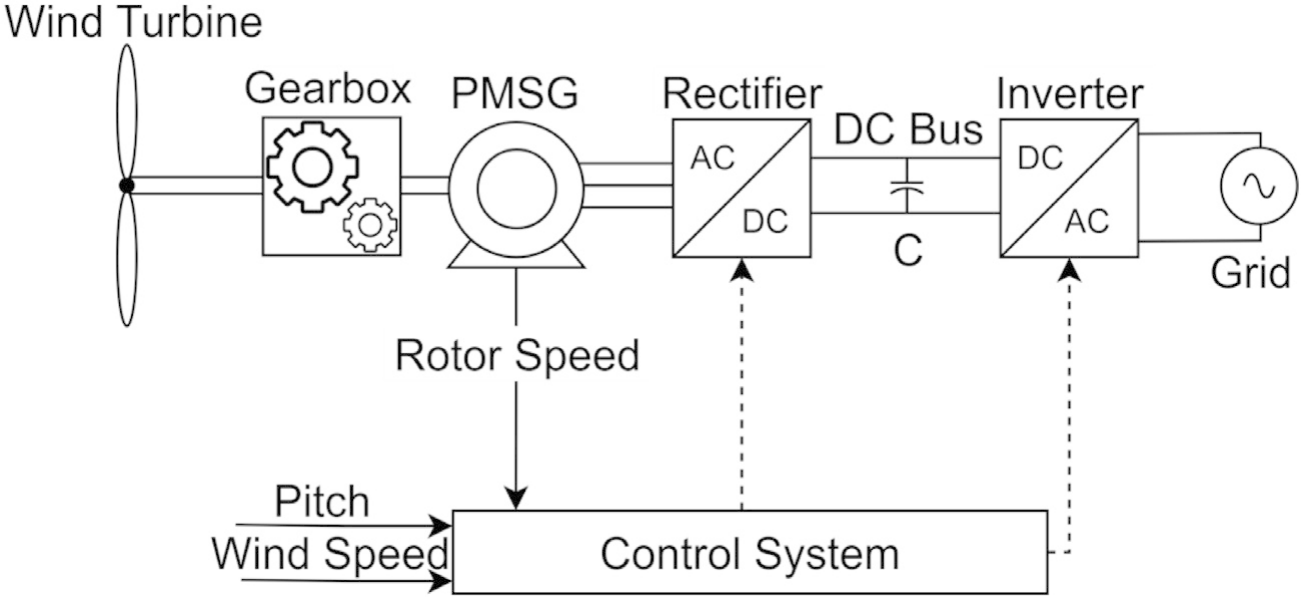
Block diagram of a PMSG wind turbine system.

Fig. 8 presents a block diagram of a typical PMSG wind turbine system and its control interfaces, as commonly found in wind farm applications. The system operates as follows: the wind turbine, representing the blades that capture the kinetic energy of the wind, is mechanically coupled to a gearbox. The gearbox adjusts the rotational speed from the turbine to match the optimal operating speed of the PMSG, improving energy conversion efficiency. An encoder, coupled to PMSG, provides a signal corresponding to the rotor speed to the control system. The generator connects in a three-phase configuration to a power rectifier. This rectifier couples to the inverter through a DC bus equipped with a capacitive filter. The converter interfaces with the electrical grid. The control system communicates with both converters to regulate the system's operation. The control system receives the blade pitch angle and wind speed as inputs.

To simulate a wind turbine, the electrical grid supplies power through a WEG CFW500 frequency inverter, which drives a squirrel cage induction motor. This motor acts as the primary machine for the PMSG, providing torque. The CFW500 enables precise speed and torque control of the three-phase induction motor, allowing accurate emulation of the dynamic behavior of a single wind turbine [12].

The CFW500 performs torque control, with the reference torque provided by the Imperix Control B-Box, which is a high-performance real-time control platform specifically designed for the development, prototyping, and implementation of power electronic systems and control algorithms. It integrates a powerful FPGA and DSP-based processing architecture, allowing for deterministic execution of complex control strategies with low latency. The B-Box features flexible analog and digital I/O interfaces, as well as compatibility with industrial communication protocols such as CAN and EtherCAT, enabling the integration with sensors, actuators, and other test bench components [13]. Its programmability and reliability make it a valuable tool for hardware in the loop (HIL) testing and experimental validation of advanced control techniques in power electronics and electrical machines.

**Fig. 9:**
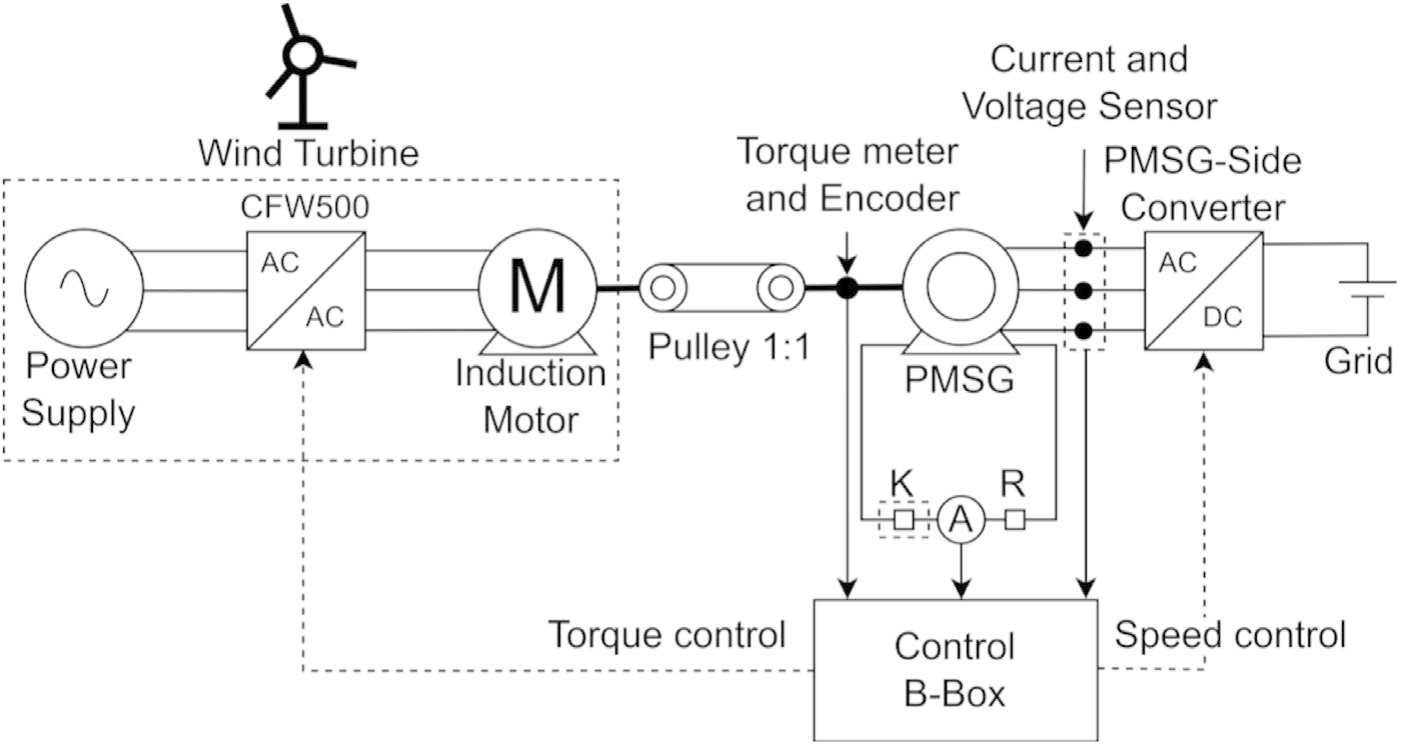
Block diagram of the PMSG system built on the test bench.

Fig. 9 shows the block diagram of the test bench that includes the PMSG. The solid arrows represent interactions between blocks or show component placement. Dashed arrows represent control signals exchanged between blocks. The first group of blocks, comprising the power supply, converter, and induction motor, simulates a wind turbine. The induction motor acts as the primary machine, providing mechanical torque via a pulley that functions as a gearbox, with a unity gear ratio, to drive the PMSG. A torque meter and an encoder are installed between the PMSG and the pulley, which transmit torque and shaft angle information to the control system, referred to as the Control B-Box, via a CAN [14]. The PMSG includes two derivation points used for fault insertion. These points connect to a contactor (represented by K), a current transducer, and the fault resistance (represented by R), all of which interface directly with the B-Box. The PMSG is connected to a converter, which is in turn connected to the electrical grid. Current and voltage sensors placed between them provide measurements to the Control B-Box. Finally, the Control B-Box references the torque for the converter and performs the speed control of the converter.

Table 4 shows all the components of the PMSG system and their corresponding models and descriptions. The power supply used in this system has a nominal voltage of 800 V, nominal current of 25 A, and nominal power of 6 kW [15].

**Table 4:** Bench PMSG system description.

Name	Model	Description
Power supply	-	Provides electrical energy from the grid.
Converter	CFW500B16P0T2DB20G2	Controls the torque provided by the primary machine.
Induction motor	WEG W22 Premium 4p L100L 3.7kW	Primary machine for mechanical torque.
Pulley 1:1	Custom made by Equacional	Mechanical torque transferred from the primary machine to the generator.
Torque meter	HBM T22 [16]	Sends torque data to the Control B-Box.
Encoder	BRT50 [17]	Sends generator position to the Control B-Box.
PMSG	Custom made by Equacional	Electrical power generator.
Current sensor	Native PEB8038 sensor (for Iabc measurement)	Current signal collector for the control system.
Voltage sensor	Native PEB8038 sensor (for Vdc) DIN 800 V (for Vabc)	Voltage signal collector for the control system.
Converter	PEB8038	Converts alternate current to direct current.
Grid	IT6006–800–25	DC Power Supply.
K	CJX2 3210Z	DC contactor for fault insertion.
R	-	Fault resistance.
A	DIN 50A	Current transducer.
Control B-Box	PEB8038	Control system of the PMSG system.

Sensors directly connected to the Control B-Box acquired electrical signals from the test bench. PEB 8038 modules, specifically designed for accurate acquisition of high-voltage and high-current signals, measured the PMSG current and the DC-link voltage. A DIN rail-mounted voltage sensor rated for 800 V monitored the three-phase voltage of the PMSG, while a DIN-mounted current sensor rated for 50 A acquired the fault current. The selection of sensors prioritized reliability and seamless integration with the B-Box hardware, ensuring accurate measurements during both steady-state and transient conditions. The configuration of gain settings optimized the signal range, and the 14-bit resolution of the analog-to-digital converter ensured the fidelity required for later analysis. The torque was measured using the T22 Torque Transducer, which can measure rated torque from 0.5 Nm to 1 kNm [16]. The encoder BRT50 [17] acquired the rotary measurements.

### 4.1 Photographic overview of the test bench and laboratory

**Fig. 10:**
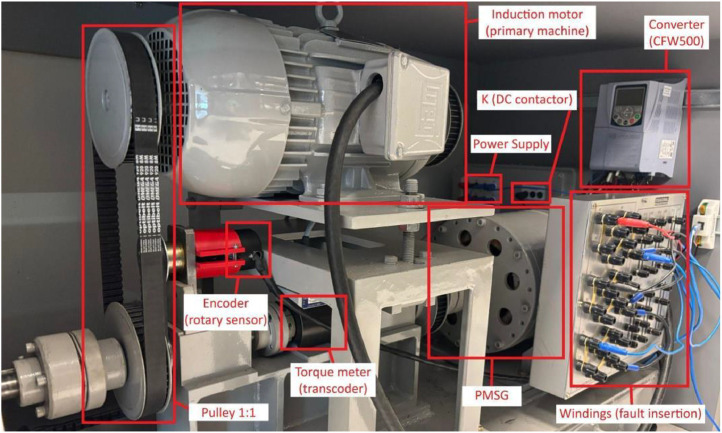
Test bench of the PMSG system.

Fig. 10 summarizes the PMSG setup on the test bench, with each component identified and enclosed in red-labeled squares.

**Fig. 11:**
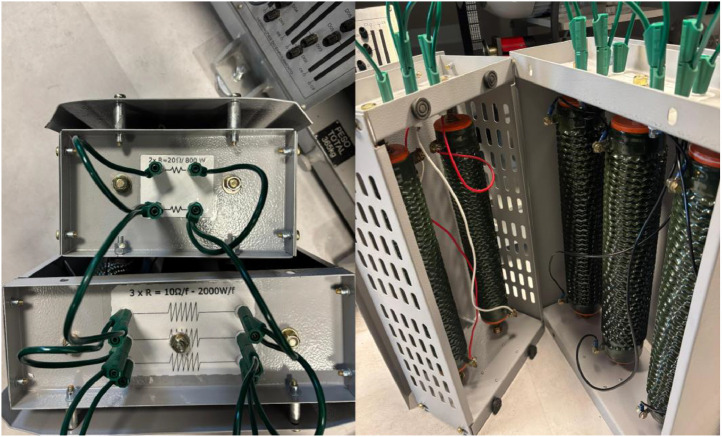
Resistance set in parallel configuration for the PMSG system.

Fig. 11 shows the resistor assembly connected in parallel, which results in the desired fault resistance. Parallel configuration also improves power dissipation by distributing the thermal load across multiple resistors. This resistance is connected between the PMSG and the Control B-Box and is inserted into the circuit via contactor K.

**Fig. 12:**
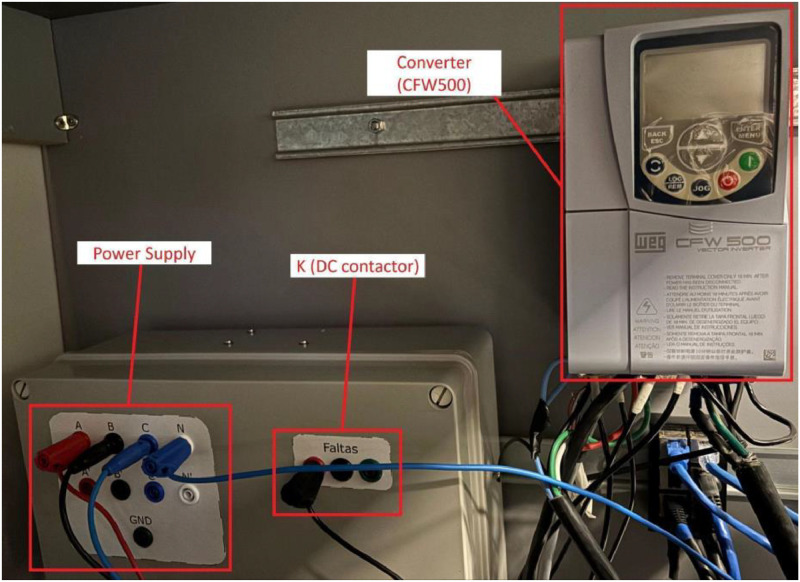
Close view of the converter, power supply, and the DC contactor.

Fig. 12 provides a closer view of the converter, power supply, and contactor K assembly. Switching contactor K directly inserts the fault into the derivations of the PMSG windings. The power supply feeds the converter, which is connected to the primary machine.

**Fig. 13:**
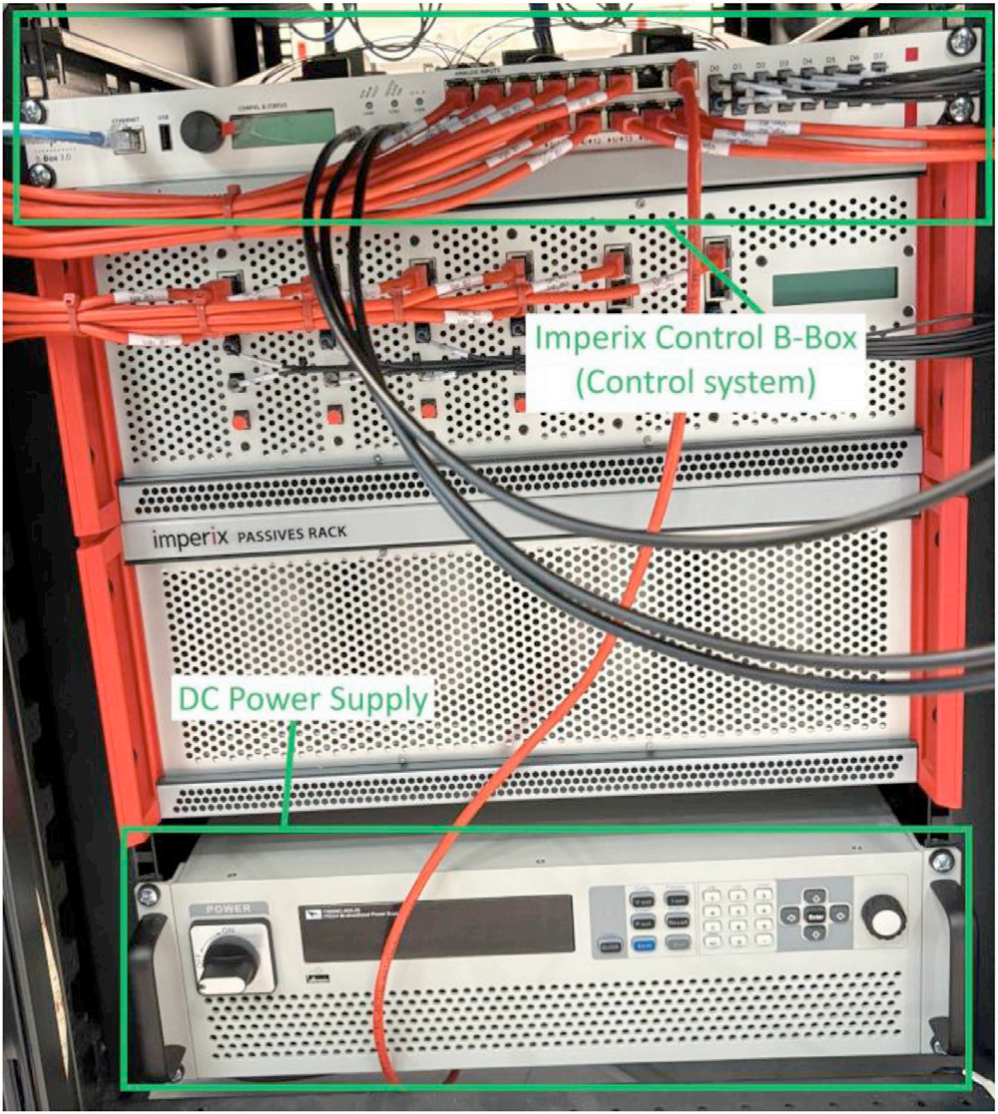
Imperix control B-Box and DC Power Supply from the PMSG bench.

Fig. 13 shows the Imperix Control B-Box positioned at the top and the DC power supply at the bottom. The experimental test bench setup requires the DC supply to be set to 600 V to provide adequate conditions for the converter to synthesize the control voltages. This value reflects the nominal voltage of the PMSG, with an additional margin to support proper modulation. When the supply displays positive power, it indicates that energy flows into the PMSG. Conversely, a negative power reading means the PMSG is sending energy back to the supply. Each red cable connected to the B-Box corresponds to a sensor input, while the gray cables on the right-hand side of the image represent the switching commands for the converter. These gray cables are optical, ensuring proper isolation and signal integrity.

**Fig. 14:**
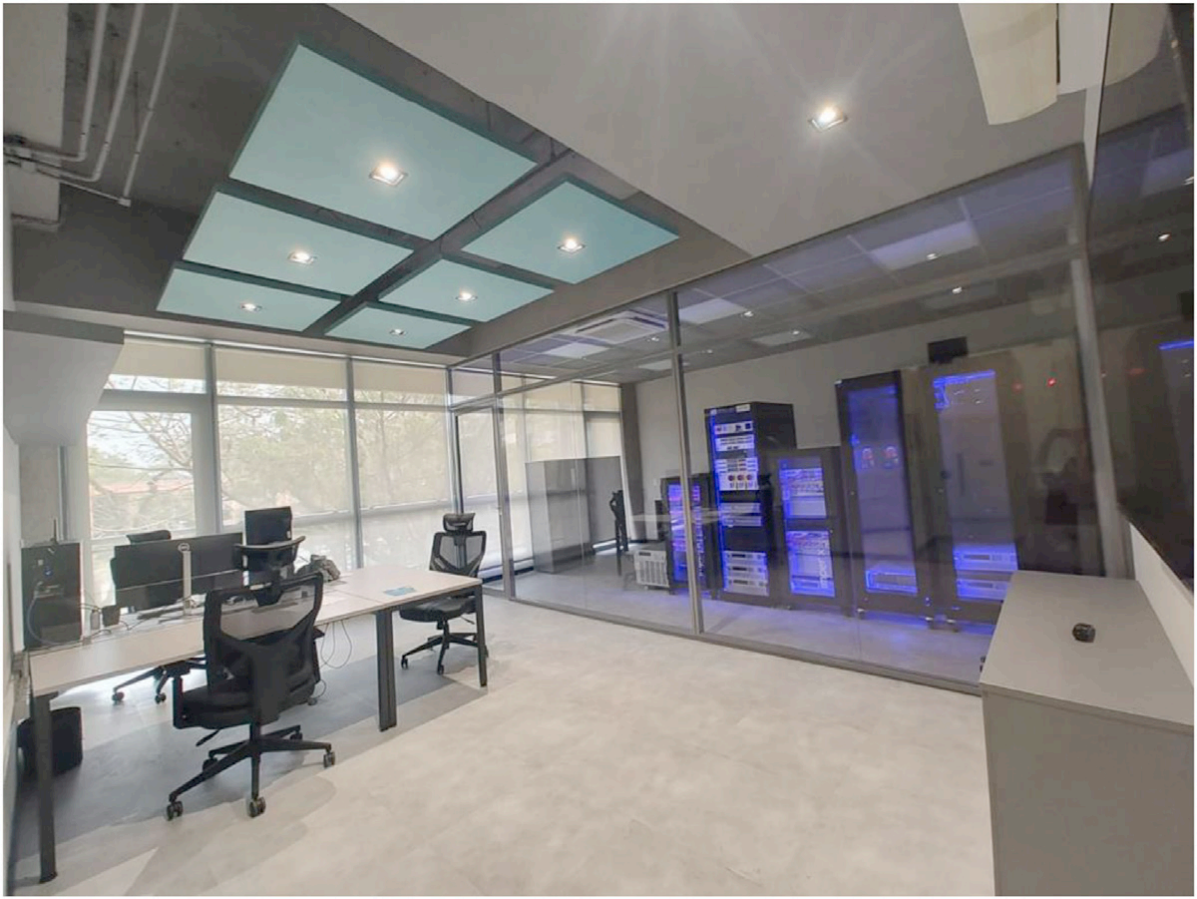
InnovaPower - real-time simulation laboratory.

The experimental test bench is located in the Real-Time Simulation Laboratory at the InnovaPower Hub, University of São Paulo (USP) [18]. Fig. 14 presents the laboratory environment in which the experimental procedures took place. This laboratory has a goal of supporting InnovaPower’s projects with advanced infrastructure for research validation that enables real-time analysis of various components with support for both HIL and Power-HIL validation.

The InnovaPower Hub currently hosts 13 R, D&I (Research, Development, and Innovation) programs, comprising 11 research projects and 2 infrastructure initiatives. This center involves approximately 180 active researchers, including professors, postdoctoral fellows, PhD and MSc candidates, and undergraduate students. The laboratory infrastructure also accommodates different electrical machines, such as PMSGs and Doubly-Fed Induction Generators (DFIGs), making it particularly suitable for studies related to renewable energy systems and fault diagnosis.

### 4.2 Script adopted for data generation

Following the introduction of the laboratory environment and the experimental setup, this section presents the method adopted for data acquisition. An adapted Python script collected data for each of the analyzed cases through the Imperix Control B-Box, acting as the interface between the hardware components and the control logic. This script includes commands referencing previously introduced equipment, such as the CFW500 inverter, facilitating the readability.

**Fig. 15:**
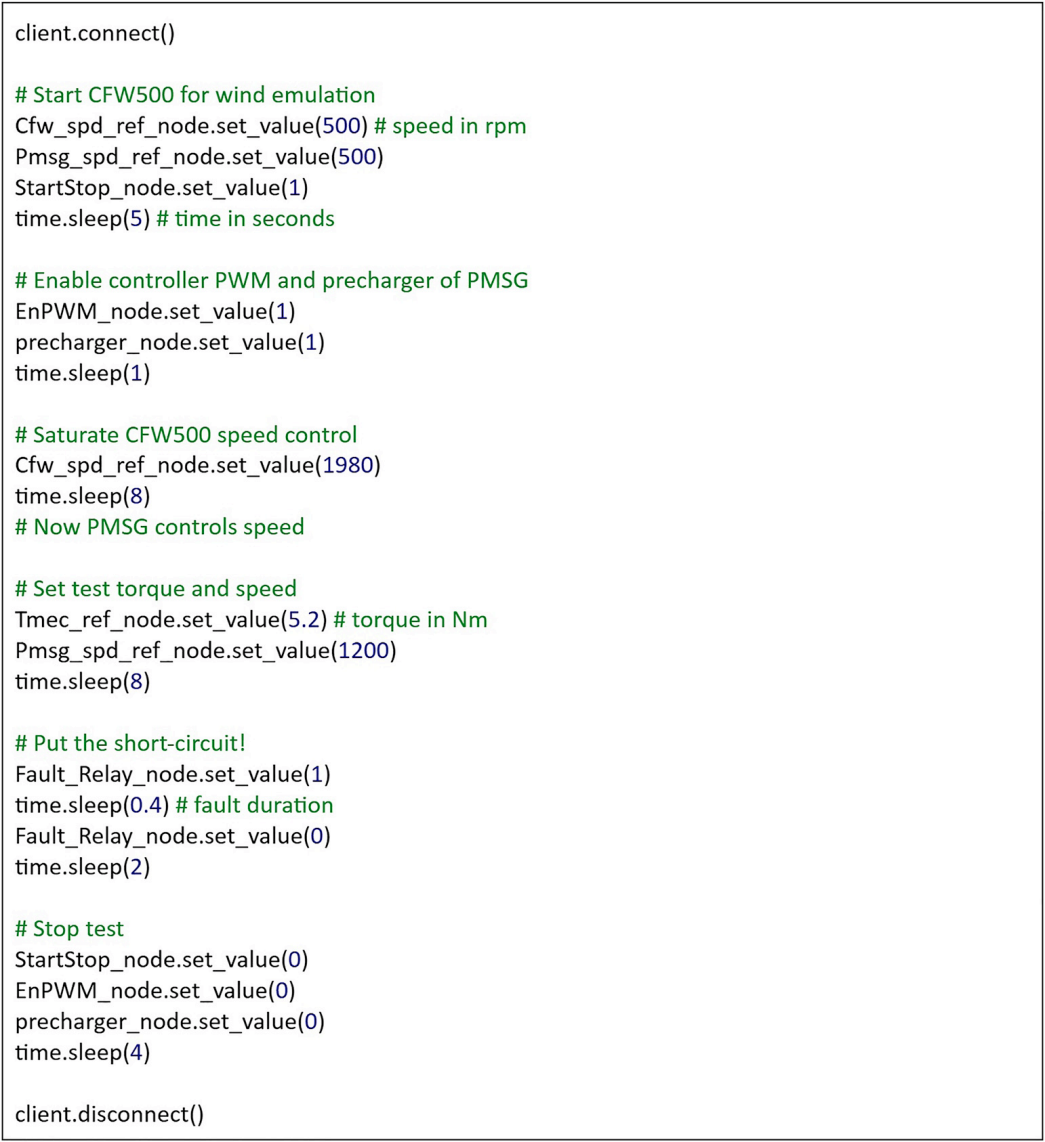
Simplified python script for test execution.

Fig. 15 shows the Python script used to perform the experimental tests. Table 5 describes each node in the script. Each node refers to a variable in the OPC UA server associated with a specific hardware control point, such as the speed reference or the fault relay command.

**Table 5:** Variables python script description.

Node name	Node description
Cfw_spd_ref_node	CFW500 speed reference adjustment in rpm.
Pmsg_spd_ref_node	Speed reference adjustment for PMSG control in rpm.
StartStop_node	Command to start (1) and stop (0) the CFW500.
EnPWM_node	Command to enable (1) and disable (0) the PWM of the PMSG control.
precharger_node	Command to enable (1) and disable (0) the DC-link precharger.
Tmec_ref_node	Mechanical torque adjustment for PMSG control in Nm.
Fault_Relay_node	Contactor command to put (1) and remove (0) short-circuit.

A Samsung Galaxy Book3 Ultra executed the script 225 times, generating one data file per run. The laptop has a 13th-generation Intel Core i7 processor, 32 GB of RAM, and a dedicated NVIDIA GeForce RTX 4050 GPU.

After each execution, we manually verified all collected variables to ensure data integrity and quality. The wide variety of possible acquisition anomalies made automation impractical, as scripted validation could not account for all potential failure modes. Manual inspection prevented the inclusion of corrupted data and ensured that the dataset accurately represented the intended short-circuit scenarios. When an issue appeared, the test was repeated.

Common issues observed during these verifications included transient disturbances in current sensor readings, especially when measurements neared the sensor’s lower scale limit, and delays in CAN communication. In the latter case, the control occasionally processed outdated signals, causing the integrator to respond excessively. This effect introduces disturbances in the system’s behavior that could be misinterpreted as part of the short-circuit response. Although such phenomena may occur in real-world applications, the dataset excludes excessively distorted responses to maintain its focus on short-circuit characterization.

All tests were conducted under supervision, with safety mechanisms in place to avoid equipment damage during fault conditions.

The test script requires the CFW500 inverter speed to be saturated to 1980 rpm to ensure that its control would not interfere with the PMSG control under any of the operational conditions considered. This value is necessary because it exceeds the maximum test speed of 1800 rpm, providing a 10 % margin that helps maintain control effectiveness even in the presence of transient oscillations during dynamic conditions.

A 400 ms time window ensures at least 10 cycles with the PMSG under fault. While this duration could theoretically be shortened for cases with lower rotational speeds, dynamically adjusting the fault duration for each test would introduce unnecessary complexity and increase the likelihood of errors, especially since all parameters were manually tuned.

Sleep intervals are necessary to ensure the machine reaches steady-state conditions before each test phase. These intervals were empirically determined and varied to minimize total test time while maintaining reliability. Optimizing these pauses further was deemed unnecessary, as it would offer negligible benefits compared to the time required for such tuning.

## Limitations

The dataset includes measurements from only one low-power machine. Additional datasets may be required to achieve satisfactory performance in the proposed applications presented in Value of the Data. It was not possible to perform tests and acquire data on high-power machines because of physical installation and safety limitations. The sensor readings are subject to minor measurement errors because of the inherent limitations in instrumentation precision. Data storage constraints and time limitations restricted the combinations of speed and torque to three different values each. Fault intensity and duration were limited to protect the equipment’s integrity. The severity and higher risk of permanent machine damage of inter-phase faults led to their exclusion from consideration. These faults tend to be more easily detected, which also contributed to their exclusion in this work.

## Ethics Statement

Our research adheres to the ethical requirements for publication in Data in Brief, does not involve human or animal subjects, and no data has been collected from social media platforms.

## CRediT authorship contribution statement

**Rafael Noboro Tominaga:** Conceptualization, Methodology, Software, Validation, Formal analysis, Investigation, Data curation, Writing – original draft, Visualization. **Santiago Silveira Barbosa:** Investigation, Writing – original draft, Visualization. **Luan Andrade Sousa:** Methodology, Software, Validation, Investigation, Resources. **Angelo dos Santos Lunardi:** Software, Validation, Investigation. **Rodolfo Varraschim Rocha:** Conceptualization, Methodology, Software, Validation, Investigation. **Sérgio Luciano Ávila:** Conceptualization, Writing – review & editing, Supervision. **Bruno Souza Carmo:** Conceptualization, Writing – review & editing, Resources, Project administration, Funding acquisition. **Renato Machado Monaro:** Conceptualization, Methodology, Software, Investigation, Resources. **Maurício Barbosa de Camargo Salles:** Conceptualization, Resources, Project administration, Funding acquisition.

## Data Availability

GithubPMSG-3phase-Dataset (Original data).
